# A low-cost uterine balloon tamponade for management of postpartum hemorrhage: modeling the potential impact on maternal mortality and morbidity in sub-Saharan Africa

**DOI:** 10.1186/s12884-017-1564-5

**Published:** 2017-11-13

**Authors:** Tara Herrick, Mercy Mvundura, Thomas F. Burke, Elizabeth Abu-Haydar

**Affiliations:** 1 PATH, Market Dynamics, 2201 Westlake Ave, Suite 200, Seattle, WA 98121 USA; 2 PATH, Devices and Tools, Seattle, WA USA; 3 Division of Global Health and Human Rights, Department of Emergency Medicine, Massachusetts General Hospital, Boston, MA USA; 4 Harvard Medical School, Boston, MA USA; 5 Harvard T.H. Chan School of Public Health, Boston, MA USA

**Keywords:** Maternal mortality, Postpartum hemorrhage, Uterine balloon tamponade, Sub-Saharan Africa, Health impact modeling, Low-income countries

## Abstract

**Background:**

Postpartum hemorrhage (PPH) is the leading cause of maternal deaths worldwide. This study sought to quantify the potential health impact (morbidity and mortality reductions) that a low-cost uterine balloon tamponade (UBT) could have on women suffering from uncontrolled PPH due to uterine atony in sub-Saharan Africa.

**Methods:**

The Maternal and Neonatal Directed Assessment of Technology (MANDATE) model was used to estimate maternal deaths, surgeries averted, and cases of severe anemia prevented through UBT use among women with PPH who receive a uterotonic drug but fail this therapy in a health facility. Estimates were generated for the year 2018. The main outcome measures were lives saved, surgeries averted, and severe anemia prevented.

**Results:**

The base case model estimated that widespread use of a low-cost UBT in clinics and hospitals could save 6547 lives (an 11% reduction in maternal deaths), avert 10,823 surgeries, and prevent 634 severe anemia cases in sub-Saharan Africa annually.

**Conclusions:**

A low-cost UBT has a strong potential to save lives and reduce morbidity. It can also potentially reduce costly downstream interventions for women who give birth in a health care facility. This technology may be especially useful for meeting global targets for reducing maternal mortality as identified in Sustainable Development Goal 3.

## Background

Maternal mortality remains high in low- and middle-income countries despite a 43% drop in maternal death rates worldwide since 1990 [1, 2]. As the world seeks to achieve the Sustainable Development Goal (SDG) 3 maternal health targets—to reduce the global maternal mortality ratio to less than 70 per 100,000 live births by 2030 [3]—a key challenge will be to address the main causes of maternal death by increasing access to effective innovations. Postpartum hemorrhage (PPH) is the leading cause of maternal mortality. It contributes to 115,000 maternal deaths a year [2, 4], with 99% of these deaths occurring in low-resource settings, including sub-Saharan Africa (SSA). Defined as vaginal bleeding in excess of 500 mL within 24 h of delivery [5], PPH is dangerous and life-threatening and can also lead to long-lasting health effects, including severe anemia. Additionally, those who survive severe PPH (greater than 1000 mL of blood loss) [6] are more likely to die in the following year from related complications, ranging from heart conditions to organ failure to depression [7, 8].

The most common cause of PPH is uterine atony, or failure of the uterus to contract and retract following delivery of the baby. Treatment for atony is clearly stated in global recommendations and follows a well-defined stepwise approach, including drugs and mechanical interventions followed by surgery as a last resort [5, 9]. Nonetheless, access to these key interventions is often lacking [10]. In cases where bleeding persists, the woman is at grave risk of dying unless she can get to a health facility equipped with obstetric surgical care. Obstetric surgical capabilities and emergency transportation are limited in low-resource settings, highlighting the need for effective treatments at the point of care.

In 2012, the World Health Organization (WHO) recommended use of the uterine balloon tamponade (UBT) for treatment of uterine atony when uterotonic drugs are unavailable or ineffective [5]. UBT is a nonsurgical intervention that can be administered by trained health care providers at the point of care. The balloon is inserted into the uterus and filled with clean water [11], and effective tamponade occurs rapidly to stop hemorrhage. Published case series and systematic reviews have shown that UBT devices are safe and effective, with a success rate of 85% to 95% for treating PPH unresponsive to medical management [11–25]. Nonetheless, UBT is still widely underutilised and unavailable in low- and middle-income countries, largely because commercial devices are prohibitively expensive, ranging from $US125 to $350 for one-time use. Recent efforts to develop low-cost UBTs, which could cost approximately 10% of current prices, would provide opportunities to expand access to lifesaving treatments [26, 27].

Low-cost UBTs fall into two categories. The first is the condom catheter, which is assembled at the point of care using a catheter, a condom tied to the catheter, and a syringe for filling the condom. An example is the Every Second Matters UBT, a device designed by Massachusetts General Hospital that includes an illustrated set of user instructions as well as the needed components. A second type of device is manufactured in South Africa by Sinapi Biomedical and is a fully assembled UBT designed as an open-gravity fill system. Both types of UBTs are intended for use by trained health providers for the treatment of postpartum hemorrhage.

This study aimed to quantify, through modeling, the potential health impact (morbidity and mortality reductions) that a low-cost UBT could have on women suffering from uncontrolled PPH in SSA in the year 2018. It sought to estimate how increasing use of the UBT may save lives, prevent severe anemia, and reduce the need for surgical measures. The modeling results can be used to inform investment decisions and policies related to development and deployment of a low-cost UBT.

## Methods

The Maternal and Neonatal Directed Assessment of Technology (MANDATE) model, developed by the Research Triangle Institute, was used to estimate the potential health impact of introducing a low-cost UBT device in SSA. The MANDATE model enables users to estimate the potential number of lives saved based on the availability, utilization, and efficacy of technologies in various care settings for select maternal and neonatal disease areas [28]. This publically available model was selected because it can be used to evaluate pipeline technologies to manage and prevent PPH. The model was developed with funds from the Bill & Melinda Gates Foundation.

The MANDATE baseline PPH model first determines the number of live births in SSA that occur in home, clinic, and hospital settings. For each of these settings, the model estimates the population that suffers from PPH due to atonic uterus, retained placenta, and lacerations. In addition, the model includes underlying assumptions on the proportion of women who receive existing PPH prevention, diagnostic, and treatment interventions. Finally, the model includes maternal case fatality rates to estimate the number of deaths due to PPH. A detailed overview of the MANDATE PPH model framework and a summary of the inputs has been previously published [29]. Some of the key data sources used in the MANDATE model include United Nations population estimates for live birth rates, Demographic and Health Surveys data for delivery rates by setting, and mortality data from the WHO and the Institute for Health Metrics and Evaluation [30].

Using the baseline MANDATE postpartum hemorrhage model framework generates maternal mortality estimates that are consistent with the literature [29]. Table 1 shows some of the key MANDATE model baseline inputs used for this modeling exercise. All model inputs are based on 2018, chosen to represent a year when a low-cost UBT could potentially be used at scale. In addition, all model inputs are based on use in SSA, which was selected for focus because of the high burden of treatable PPH.

**Table 1:** Key MANDATE model inputs, sub-Saharan Africa, for the year 2018^a^

Number of live births	34,806,654
% of births that occur in the hospital	15%
% of births that occur in the clinic	35%
% of births that occur in the home	50%
% of deliveries resulting in PPH in the hospital	8%
% of deliveries resulting in PPH in the clinic	10%
% of deliveries resulting in PPH in the home	12%
% of PPH due to atonic uterus	90%

^a^The MANDATE model has undergone a number of updates. These assumptions align with the assumptions in the March 2014 version of the model

To estimate the impact of a low-cost UBT device, a literature review was performed to determine key UBT MANDATE model inputs. We focused on determining peak assumptions for penetration, utilization, and efficacy. Penetration is defined as the availability of the innovation in a given setting. Utilization is defined as the rate of appropriate use of the innovation given that it is available. Efficacy is defined as the ability of the innovation to achieve a successful outcome.

The UBT estimates derived from the literature review were input into the baseline MANDATE model for home, clinic, and hospital settings. In this analysis, we assumed that the UBT would be available to women who suffer from PPH due to uterine atony but fail uterotonic drug treatment in clinic and hospital settings. Because the model defines the home setting as having limited availability of skilled providers, we did not assume that the UBT would be available for use in this setting. We also did not estimate the impact of the UBT for other potential indications, such as cesarean operation, placenta previa and accreta, and postabortion care. Using the UBT in situations where uterotonics are ineffective or unavailable is consistent with WHO recommendations, although country programs have been slow to introduce UBT for management of PPH.

Next, we estimated lives saved, severe hemorrhage cases averted, and surgeries averted in SSA in 2018 if a UBT were available at scale. By matching penetration rates of the UBT to existing use of uterotonic drugs in the model, we modeled a situation where the UBT would be available to women who fail uterotonic drug treatment in the clinic and hospital settings. The model defines clinics across all countries as having some availability of skilled providers and access to basic health medicines and technologies, and it defines hospitals as having skilled providers available and generally having cesarean and surgical capabilities. We assumed that a low-cost UBT innovation, which is estimated to be at least ten-fold less expensive than existing commercially available UBTs, has potential to increase availability in resource-constrained settings. It should be noted that the baseline MANDATE model does not include availability of the UBT in clinics and hospitals in SSA. This low availability/coverage rate assumption is consistent with a review of SSA country essential commodities lists, United Nations Children’s Fund Supply Division, and United Nations Commission on Life-Saving Commodities for Women and Children, which do not list the UBT, and with reports from the field, which have found that UBTs are scarce and typically limited to referral hospitals and health clinics in some countries that are piloting UBT introduction [20].

Finally, we performed sensitivity analysis on the efficacy variable for women who suffer from severe hemorrhage. More specifically, we sought to understand how lives saved would change with three different efficacy assumptions for women with severe hemorrhage. The assumptions we used represent a best case (optimistic) scenario, a more realistic (base) scenario, and a worst case (pessimistic) scenario.

Although substantial data are available on UBT effectiveness and routinely show an effectiveness ’between 85% and 95%, few studies have specifically evaluated effectiveness for women with severe PPH. In this analysis, we adopt the standard definition for severe PPH as loss of more than 1000 mL of blood. The scarcity of data for women with severe PPH is likely due in part to the fact that estimating precise amounts of blood loss during hemorrhage is challenging, especially in resource-constrained settings. We also ran a scenario where we increased the efficacy of UBT in the nonsevere patient population to 95%. Table 2 summarizes our key UBT inputs, definitions, and assumptions.

**Table 2:** Key UBT model inputs, definitions, and assumptions for the year 2018

Input	Definition	Assumptions		
		Home	Clinic	Hospital
Penetration	Availability of the UBT in a given setting	0%	60%	80%
Utilization	Rate of appropriate use of the UBT given it is available	0%	85%	85%
Efficacy (<1000 mL)	Ability of the UBT to achieve a successful outcome for blood loss	85%	85%	85%
Efficacy (>1000 mL)		60% (P)^a^	60% (P)	60% (P)
		70% (B)^a^	70% (B)	70% (B)
		80% (0)^a^	80% (0)	80% (0)

^a^Base, optimistic, and pessimistic assumptions are labeled B, O, and P, respectively

## Results

**Fig. 1:**
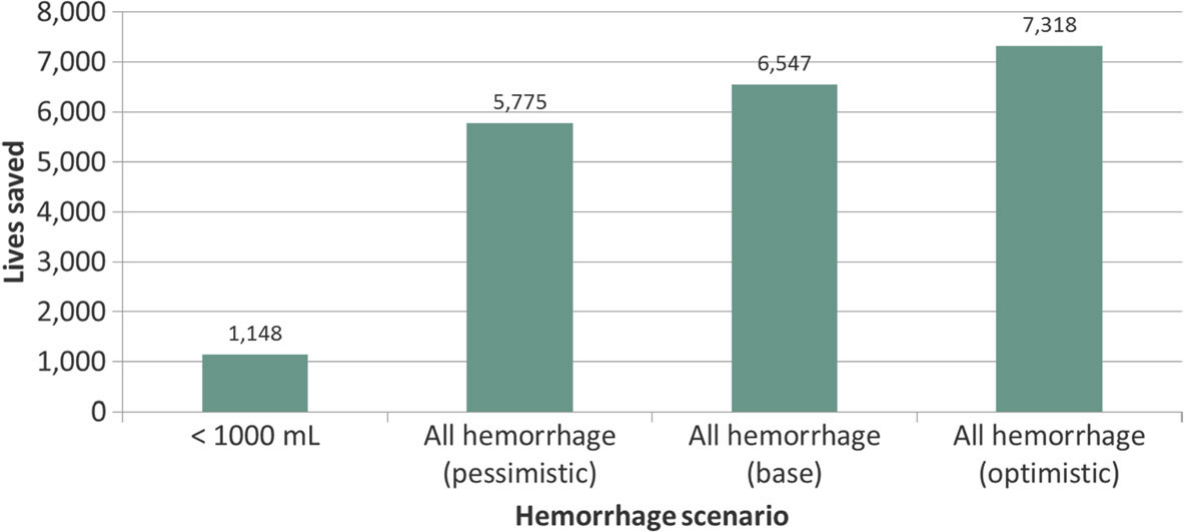
Estimated lives saved with use of a uterine balloon tamponade under four modeling scenarios. *See Table 2 for description of assumptions underlying each scenario. An estimated 6547 lives are saved in sub-Saharan Africa in 2018 under the base scenario, equivalent to an 11% reduction in maternal mortality due to postpartum hemorrhage. This scenario assumes that UBT has an efficacy of 70% for stopping hemorrhage

Our base scenario analysis found that approximately 6547 lives could be saved in SSA in 2018 if the UBT is used for women with both severe and nonsevere PPH in health facilities and hospitals (Fig. 1). This is an 11% reduction in maternal mortality in SSA due to PPH. The estimated number of lives saved increases by approximately 800 in the optimistic scenario and decreased by 800 in the pessimistic scenario. In addition, when we increased the efficacy variable to 95% in the nonsevere patient population, we saw a 10% increase in lives saved (data not shown).

Interestingly, if the UBT were used only in the nonsevere patient population (those losing less than 1000 mL of blood), the estimated impact would be more modest, saving approximately 1148 lives. However, in this population, the UBT would also avert an estimated 5287 cases of severe hemorrhage and 10,823 surgeries, largely hysterectomies, reducing morbidity and downstream costs. Although severe anemia is not estimated in the model, published data suggest that approximately 12% of those who suffer from severe hemorrhage will also suffer from severe anemia [7]. We calculated that 634 cases of severe anemia could be averted.

## Discussion

Our findings show that expanding access to low-cost UBTs in SSA can potentially result in a substantial health impact by reducing maternal mortality. To our knowledge, our analysis is the first peer-reviewed study to evaluate the potential morbidity and mortality impact of low-cost UBTs. A previous analysis, in the grey literature, evaluated the health impact and cost-effectiveness of several interventions to prevent or manage PPH and estimated that use of a UBT could have averted a significant number of PPH deaths in 2010 in Africa [31]. The lives saved estimates generated in that analysis for Africa are ten-fold higher than what we report. The difference could be due to several factors. For example, the previous study included all African countries rather than only SSA countries and was conducted at a time when maternal mortality was higher. In addition, key assumptions, such as the continuum of care and penetration of UBT, were different. The previous study assumed that UBTs would be available at 90% of hospital births and 80% of nonhospital health facility births with skilled attendance, whereas we used a more conservative estimate based on an assumption that UBTs would be available at the same rate as existing uterotonic drugs.

More recently, the PATH-led Innovation Countdown 2030 (IC2030) project found a similar percent reduction in maternal mortality based on increased use of low-cost UBTs in health care facilities [32]. The modeling approach generated for the IC2030 project was aimed at understanding how select innovations, including a low-cost UBT, could accelerate progress toward the SDG health targets between 2015 and 2030.

The findings from our analysis can be used for several purposes. First, they can be used to advocate for continued investment in low-cost UBT innovations. This is essential to reduce maternal mortality and morbidity in low-resource settings because at present, the only option for women with refractory PPH is to be referred and potentially transferred to a hospital for hysterectomy or supportive care (blood transfusions). Unfortunately, these treatment options should be used only as a last resort because they are invasive, life-altering, costly, and only available to some women who can access hospital-based services. Introducing the UBT is lifesaving and could reduce costs related to unnecessary transfer, surgery, and supportive care [33]. In addition, data from our analysis can be combined with costing estimates to evaluate the potential cost-effectiveness of UBTs. Additional studies are needed to understand the cost and cost-effectiveness of introducing a low-cost UBT.

Our analysis has several limitations. First, although the MANDATE model uses a wide variety of data sources generated from a comprehensive literature review, we acknowledge that reliable data to estimate mortality and the impact of interventions on mortality remain limited, especially in low-resource geographic areas in SSA. The baseline model, without the introduction of the UBT, provides reasonable estimates for the number of deaths that occur today. A second limitation stems from the data sources used as inputs in modeling the impact of UBT. Primary market research with key stakeholders (e.g., buyers, ministries of health, providers) was not conducted to inform our estimates, and other inputs, such as the UBT coverage estimates, were modeled to match the estimated existing coverage of uterotonic drugs in health care facilities. A third limitation is that the modeling occurred at a regional level to estimate the impact across SSA. Country-level inputs for penetration, utilization, and efficacy were not considered.

Our estimates may have underestimated the impact of the UBT mainly because we did not evaluate the use of the UBT in the home setting. We used this conservative assumption because existing studies have focused on demonstrating that UBTs are safe and effective when administered by trained health care providers in health care facilities. There may be safety concerns related to use of devices by unskilled health workers, and this may hinder the UBT from being available for home deliveries. However, research may show promise for use of the UBT in the home setting, where approximately 50% of births occur in SSA, augmenting the impact.

## Conclusions

Our modeling work has shown that low-cost UBT solutions have the potential to save lives. Our base case modeling results estimate an 11% reduction in PPH maternal mortality attributable to use of the UBT. In addition, nearly 11,000 surgeries could be averted, further highlighting the value proposition for the UBT. We also found that most lives saved will be among women with severe blood loss. Future research should aim to capture effectiveness data in this patient population. Expanding use of the UBT for births in the home setting could further increase the health impact. In SSA, a large proportion of women continue to give birth at home, and solutions to manage and treat PPH at home are critically needed.

## Abbreviations

IC2030: Innovation Countdown 2030
MANDATE: Maternal and Neonatal Directed Assessment of Technology
PPH: Postpartum hemorrhage
SDG: Sustainable Development Goal
SSA: Sub-Saharan Africa
UBT: Uterine balloon tamponade
WHO: World Health Organization
